# Mendelian Randomization Studies of Lifestyle-Related Risk Factors for Osteoarthritis: A PRISMA Review and Meta-Analysis

**DOI:** 10.3390/ijms231911906

**Published:** 2022-10-07

**Authors:** Justin Ho, Christopher Chi Hang Mak, Vivek Sharma, Kendrick To, Wasim Khan

**Affiliations:** Division of Trauma and Orthopaedics, Department of Surgery, Addenbrooke’s Hospital, University of Cambridge, Cambridge CB2 0QQ, UK

**Keywords:** osteoarthritis, lifestyle-related risk factors, Mendelian randomization, arthritis, genetic epidemiology

## Abstract

Risk factors for osteoarthritis (OA) often exert effects over protracted time-courses. Mendelian randomization (MR) studies therefore have an advantage over conventional observational studies when studying the causal effect of long-term lifestyle-related risk factors on OA. However, given the heterogeneous design of existing MR studies on OA, the reported causal estimates of these effects remain inconsistent, thus obscuring the true extent of the biological effects of OA lifestyle-risk factors. We conducted a PRISMA systematic review and specifically included MR studies that investigated the causal effect between lifestyle-related risk factors and OA, where causal estimates for various lifestyle factors were pooled for meta-analysis. Quality of studies was assessed according to STROBE-MR guidelines. A total of 1576 studies were evaluated and 23 were included. Overall, the studies included were of high quality and had a low risk of bias. Our meta-analysis demonstrates the positive causal effect of BMI (OR_IVW-random effects_ 1.49 [1.23–1.80]) and negative causal effects of serum calcium (OR_IVW-random effects_ 0.69 [0.57–0.83]) and LDL levels (OR_IVW-random effects_ 0.93 [0.90–0.96]) on OA. Despite the heterogeneous designs and estimates of causal effects provided by various MR studies, our meta-analysis suggests that lifestyle-related risk factors in the form of BMI, serum calcium, and LDL have true biological effects on the development of OA.

## 1. Introduction

Osteoarthritis (OA) is a degenerative joint disease that ranks fifth amongst all causes of disability globally [1]. It is characterized by a loss of articular joint cartilage, thinning of the subchondral plate and the formation of osteophytes. The main problem for individuals who suffer from OA is chronic joint pain and impaired mobility that decreases quality of life—those morbid with OA are unable to perform major daily activities and 80% have movement limitations [2]. The World Health Organization predict that around 18% of all women and 10% of all men aged over 60 have osteoarthritis [3].

Whilst cartilage loss and injury are an inevitable result of ageing, not all individuals develop osteoarthritis, therefore research into modifiable lifestyle factors that predispose to the condition may provide ways of improving primary prevention and insight into putative biological pathways that may be targeted by new treatments [4]. The timeframe for risk factors impacting OA takes a long time to develop due to the etiology of the condition, therefore, observational studies in the form of case-control or prospective longitudinal studies may not always be feasible. Risk exposures such as smoking are known to result in other forms of harm if patients are subject to prolonged exposure and so are unethical.

Mendelian randomization (MR) studies are becoming increasingly prevalent and use genetic variants associated with a risk factor as instrumental variables to assess whether there is a causal effect, or if there are spurious associations due to retro-causality on a disease outcome in an observational setting [5,6]. While there exist various MR studies that study the effect of lifestyle factors on osteoarthritis, these studies differ in their design, cohort, genetic instruments, quality and therefore, the estimates of causal effect that they report. These inconsistencies demonstrate the need for a systematic review of these MR studies.

Systematic reviews for MR studies have indeed been previously conducted, such as for abdominal aortic aneurysms [7,8] but to our knowledge this is the first review to systematically summarize and meta-analyze published research that used MR to assess causal risk factors for osteoarthritis and to identify summary estimates of this causal effect between lifestyle factor exposures (BMI, serum calcium and LDL) and disease outcome.

## 2. Methods

### 2.1. Literature Search

This systematic review was performed in accordance with the 2020 updated Preferred Reporting Items for Systematic Review and Meta-Analysis Protocols (PRISMA-P) guidelines [9,10]. The study protocol was registered with PROSPERO (CRD42021266752). A comprehensive literature search was conducted using three databases: Medline via OvidSP (1946 to 31 August 2022); PubMed, EMBASE via OvidSP (1974 to 31 August 2022) and Scopus via Elsevier (all years to 31 August 2022). The full search strategy for each database, including any filters, is detailed in Appendix A. The search results were imported into Rayyan.ai for de-duplication before being screened by the inclusion and exclusion criteria [11].

The following were used as inclusion criteria for all studies screened:

1. Any studies that used MR to investigate the causal effect between any risk factor with osteoarthritis or related phenotypes;
2. Any studies that used genetic variation as a proxy (Lawlor definition) for an exposure to make causal inferences (not necessarily quantifying causal effect) regarding the effect of the instrumental variable on the outcome of osteoarthritis [12];
3. Any studies with genome-wide or phenome-wide association studies (GWAS/PheWAS) that included MR as part of their analysis;
4. Studies of all sex, age, cohorts, and ethnicities;
5. The following were used as exclusion criteria:
6. Any studies that were unrelated to risk factors on osteoarthritis outcome (MRI, imaging studies);
7. Any studies not conducted on humans;
8. Any case reports, narrative reviews, letters, editorials, opinions, incomplete manuscripts, and conference abstracts;
9. Any studies in which a full English manuscript was not accessible.

### 2.2. Data Extraction and Quality Assessment

Data from included studies were extracted (Table 1) A quality assessment was conducted based on adherence to the Strengthening the Reporting of Mendelian Randomization Studies (STROBE-MR) Guidelines [13]. The guidelines were adapted based on articles that reported quality assessment approaches utilized to document MR studies [14,15]. Upon conversion of the quality assessment score to a percentage, scores of <75%, 75–85% and >85% were considered to indicate high, medium and low risk of bias, respectively, in each of the 23 included studies (Table A1, Appendix B) [16,17,18,19,20,21,22,23,24,25,26,27,28,29,30,31,32,33,34,35,36,37,38].

### 2.3. Meta-Analysis

Data were pooled in a meta-analysis when at least 3 separate studies assessing whether there was a causal effect between the causal factor/genetic instrument with OA were identified. Meta-analyses were performed on data utilizing both the same risk factor and MR technique. Primary outcomes were defined as the association of genetic risk with osteoarthritis diagnosis or risk and were reported as OR with 95% confidence intervals. A two-sided *p*-value of <0.05 was considered statistically significant. Meta-analyses were performed using the ‘meta’ package from RStudio (Version 1.4.1717, RStudio, PBC, Boston, MA, USA). The inverse variance method with both fixed and random effects models was used alongside the DerSimonian–Laird estimator for tau^2^ in random effects analysis and the Jackson method for confidence interval of tau^2^ and tau. All ten studies were included for developing funnel plots to analyze publication bias.

## 3. Results

### 3.1. Study Characteristics

**Figure 1:**
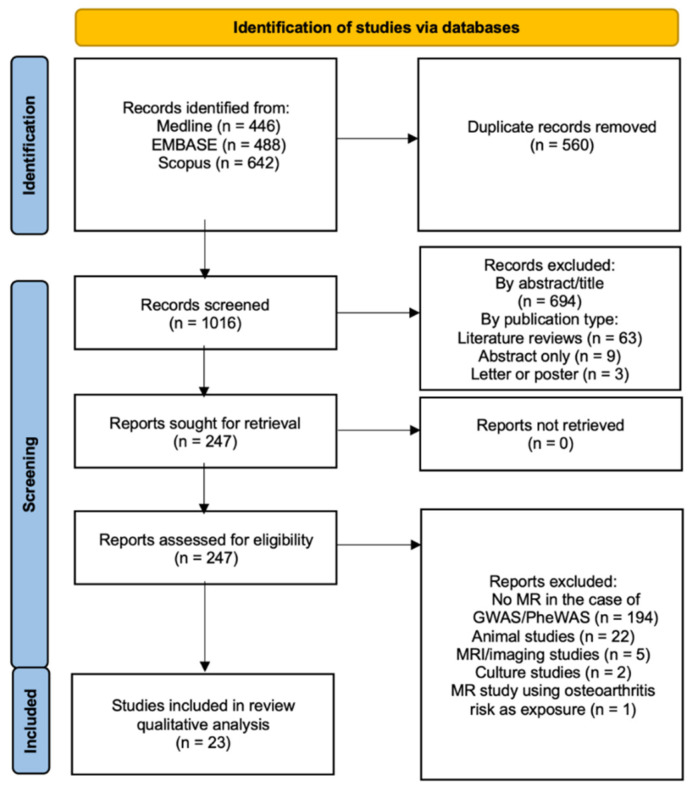
Preferred Reporting Items of Systematic Review and Meta-analyses (PRISMA) flow diagram.

The database searches returned a total of 1576 results, of which 560 duplicates were removed to yield 1016 unique records (Figure 1).

The 1576 studies were screened, and 23 studies were included (Table 1) [16,17,18,19,20,21,22,23,24,25,26,27,28,29,30,31,32,33,34,35,36,37,38]. The studies included subjects recruited from multiple datasets that were European [19,20,21,22,23,24,25,29,31,32,33,35,36,37,38], White-British [16,18,27,28,30], Swedish [17], Norwegian [26] and Icelandic [34]. The most used cohort was the UK Biobank [39] in 17 studies [16,18,20,21,22,23,24,25,27,29,30,33,34,35,36,37,38] with findings from a previous UK Biobank study replicated in one paper [17]. As the studies assessed a variety of causal factors, the number of SNPs used in studies ranged from a single SNP [26,28] to 527 SNPs [18].

The OA phenotype investigated as the outcome variable showed heterogeneity across the included studies (Table A2, Appendix B). A total of 4 studies used methods of self-report [16,21,35,37] and 17 studies used hospital diagnosis [18,19,20,22,23,24,25,27,28,29,30,31,32,33,34,36,38]. There were 2 studies that utilized both methods of diagnosis [17,26]. A total of 12 studies stratified OA by site such as knee/hip/hand [19,23,25,29,30,31,32,33,34,36,38] whereas 8 studies did not and considered osteoarthritis risk/diagnosis [16,18,21,22,24,27,28,35]. There were 3 studies that utilized the need for joint replacement surgery as the outcome variable, all considering both hip and knee replacements [17,23,26]. A total of 2 studies [25,29] also considered OA risk stratified by sex.

### 3.2. Meta-Analysis of Causal Risk Factors for Osteoarthritis

A total of 7 studies for BMI [17,18,21,23,24,27,28], 3 for serum calcium [25,29,30] and 3 for LDL [17,18,23] were selected for quantitative analysis, on the basis that there were common risk factors between studies to meta-analyze.

#### 3.2.1. Body Mass Index

**Figure 2:**
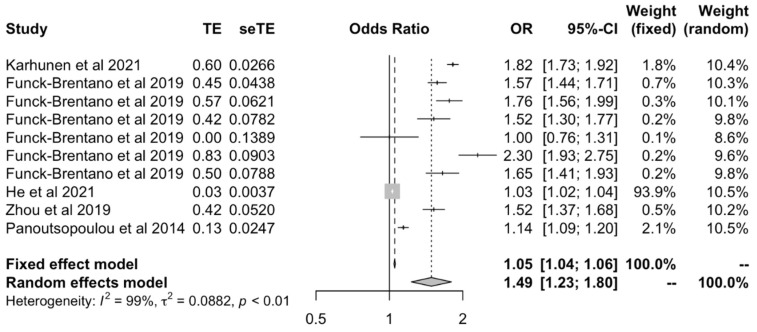
Forest plot of studies that evaluated the causal effect between BMI and all OA outcomes using values obtained by the IVW MR method.

We assessed the causal effect between BMI on all OA outcomes using values obtained only by the inverse variance-weighted method [18,23,24,27,28]; this suggested that there was a positive causal effect between BMI and all OA outcomes under both fixed effects (OR: 1.05 [1.04–1.06]) and random effects models (OR: 1.49 [1.23–1.80]) (Figure 2).

**Figure 3:**
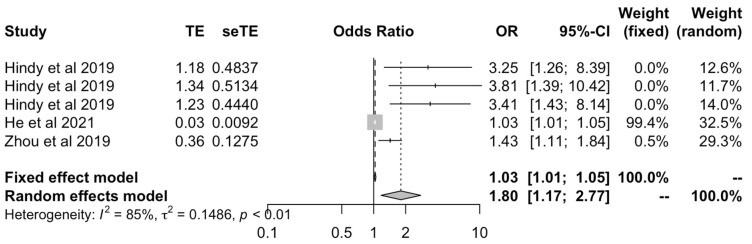
Forest plot of studies that evaluated the causal effect between BMI and all OA outcomes using values obtained by the MR-Egger methods.

**Figure 4:**
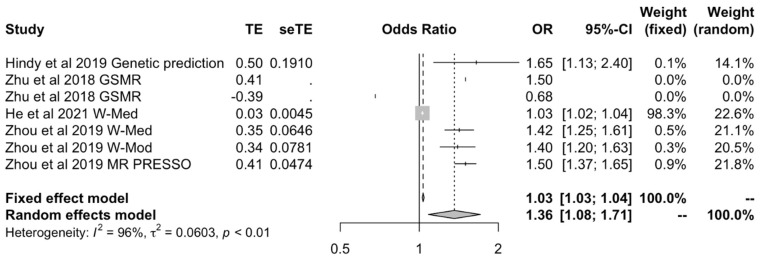
Forest plot of studies that evaluated the causal effect between BMI and all OA outcomes using values obtained by methods other than IVW and MR-Egger.

We then meta-analyzed studies that used the MR-Egger methods to include all OA outcomes [17,24,27] to assess whether results would support or rebut those from IVW data. This again showed a positive causal effect between BMI and all OA outcomes under both fixed (OR: 1.03 [1.01–1.05]) and random effects models (OR: 1.80 [1.17–2.77]) (Figure 3). Findings from the IVW and MR-Egger models were corroborated by MR studies that used various models to calculate OR and CI [17,21,24,27]. They corroborated the positive causal effect of BMI on OA when using fixed effects (OR: 1.03 [1.025–1.04]) or random effects models (OR: 1.36 [1.08–1.71]) (Figure 4).

**Figure 5:**
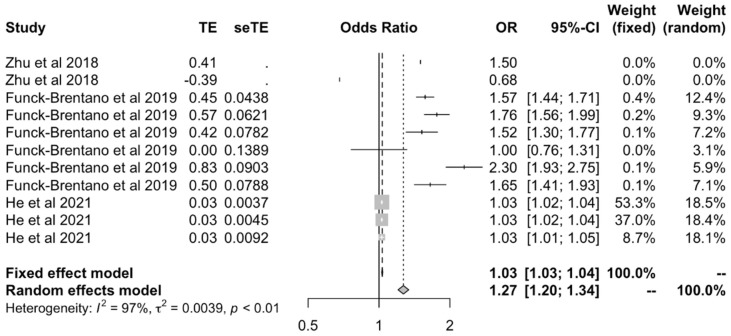
Forest plot of studies that evaluated the causal effect between BMI and all OA outcomes using values relating to European individuals.

**Figure 6:**
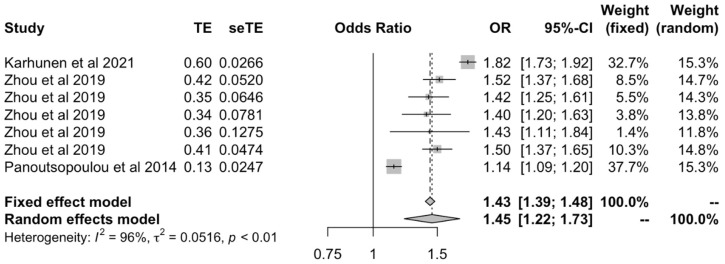
Forest plot of studies that evaluated the causal effect between BMI and all OA outcomes using values relating to White-British individuals.

Samples were then stratified and analyzed by the ethnicity of the population investigated. In the meta-analysis of studies involving European individuals [21,23,24], there was a positive causal effect between BMI and all OA outcomes under both fixed (OR: 1.03 [1.027–1.04]) and random effects models (OR: 1.27 [1.20–1.34]) (Figure 5). In the analysis of studies involving White-British subjects [18,27,28], the effect was more marked than in European populations for the fixed and (OR: 1.43 [1.39–1.48]) random effects models (OR: 1.45 [1.22–1.73]) (Figure 6).

#### 3.2.2. Serum Calcium

**Figure 7:**
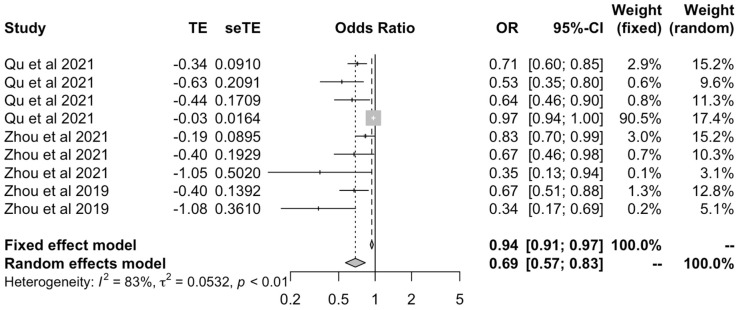
Forest plot of studies that evaluated the causal effect between serum calcium and all OA outcomes using values obtained by the IVW method.

We assessed the causal effect between serum calcium on all OA outcomes using values obtained only by the inverse variance-weighted method [25,29,30]. These illustrated a protective effect of serum calcium on OA outcomes under both fixed effects (OR: 0.94 [0.91–0.97]) and random effects models (OR: 0.69 [0.57–0.83]) (Figure 7). The I^2^ statistic reported a value of 83%.

#### 3.2.3. Low-Density Lipoproteins

**Figure 8:**
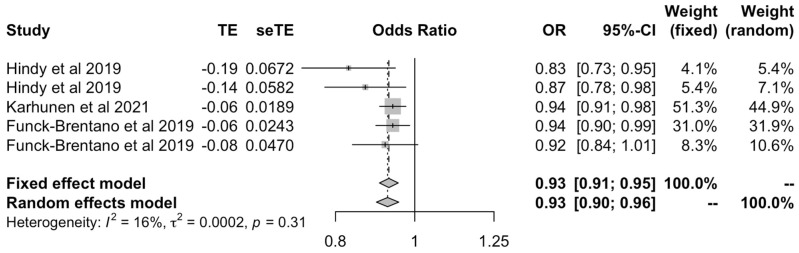
Forest plot of studies that evaluated the causal effect between LDL and all OA outcomes using values obtained by the IVW method.

The analysis of LDL cholesterol values obtained by the IVW method [17,18,23] illustrated a protective effect of LDL on OA outcomes under both fixed effects (OR: 0.93 [0.91–0.95]) and random effects models (OR: 0.93 [0.90–0.96]) (Figure 8).

**Figure 9:**
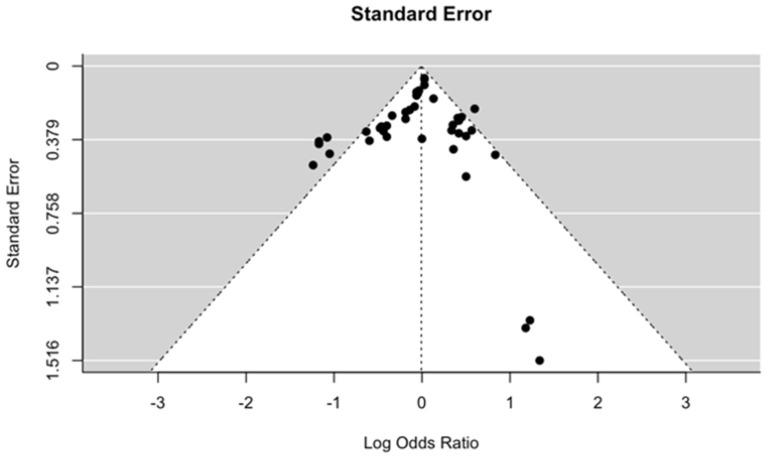
Funnel plot of the included studies, suggesting limited publication bias under fixed effects meta-regression models when the standard error was used as predictor, *p*-value 0.159.

**Figure 10:**
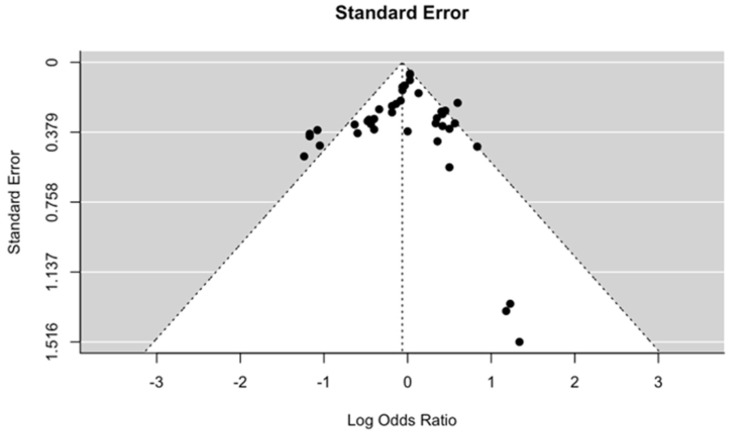
Funnel plot of the included studies, suggesting limited publication bias under mixed effects (restricted maximum likelihood) meta-regression models when the standard error was used as predictor, *p*-value 0.822.

**Figure A1:**
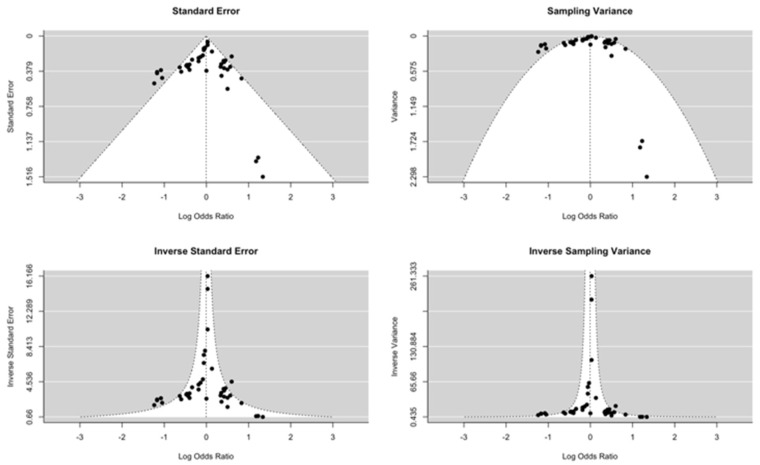
Funnel Plot for publication bias under fixed-effects sampling variance; inverse standard error and inverse sampling variance were used as predictor.

**Figure A2:**
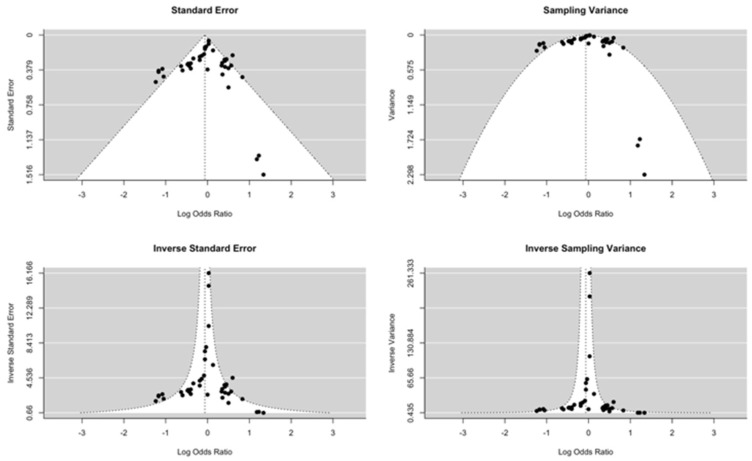
Funnel Plot for publication bias under mixed-effects sampling variance; inverse standard error and inverse sampling variance were used as predictor.

There are no standardized tools to ascertain the risk of bias in MR studies. Thus, to assess the quality of the included studies, we evaluated whether the three assumptions of MR were validated, and the method used for those validations. We constructed funnel plots to assess publication bias under both fixed effects (Figure 9) and mixed effects (Figure 10) meta-regression models when the standard error was used as the predictor. Under both conditions, there was no evidence of publication bias from the test for funnel plot asymmetry. The *p*-values for fixed and mixed effects (restricted maximum likelihood) were 0.159 and 0.822, respectively. We also constructed funnel plots when the sampling variance, inverse standard error and inverse sampling variance were used as predictor (Figure A1 and Figure A2, Appendix B).

## 4. Discussion

The main findings of this systematic review and meta-analysis were that BMI had a positive causal effect and that serum calcium and LDL levels had a negative causal effect on all outcomes of osteoarthritis. These findings were consistent with those reached in our qualitative analysis for BMI [17,18,21,23,24,27,28,35,38] and serum calcium [25,29,30]. Our findings for LDL matched those reached by Hindy et al. and Karhunen et al. [17,18], only differing from the lack of causality conclusion reported by Funck-Brentano et al. [23]. Overall, the studies included were of high quality and had a low risk of bias.

### 4.1. Body Mass Index

Our results supported those of the studies included in our systematic review that there is a direct causal effect of BMI on OA [17,18,21,23,24,27,28,35,38]. This is replicated in other genome-wide analyses [40]. OA could be in part due to systemic metabolic dysfunction secondary to high BMI, obesity and dyslipidemia [41,42]. The protective effect of education found by Karhunen et al. has been suggested to be mediated through BMI and smoking despite high uncertainty values of mediated proportion between 13% and 57% [18]. Education may have a downstream protective effect related to mechanisms such as greater engagement with healthcare practices and increased self-management, thus, reducing BMI. Funck-Brentano et al. showed that high BMI has a causal effect on increasing the risk of knee and hip OA but not hand OA [23]. Interestingly, after exclusion of genetic instruments that were also associated with BMI, other metabolic factors (HDL, triglyceride, CRP, type 2 diabetes) had no causal effect on developing OA in the same study.

BMI has its limitations as a measure of obesity as it does not account for body composition, age-related lean muscle/fat changes and sex differences so further investigation into how these factors alongside BMI affect OA risk is required [43]. Raud et al. demonstrated a dose–response relationship between BMI and knee OA [44], whilst Reyes et al. demonstrated an increased risk of hip and knee OA in overweight individuals [45]. Similar findings by He et al. from robust MR methods support the main implication that weight control would be a significant intervention in the management of OA [24]. Overall, the etiology of how BMI exerts a causal effect on OA risk has not been fully deduced. Therefore, it was postulated that whilst obesity could initially cause OA changes in the weight-bearing joints, there was a role of synergistic effects such as comorbidity, lifestyle factors and lack of mobility that resulted in further increasing the risk of severe OA symptoms.

### 4.2. Serum Calcium

For calcium, the results of our meta-analysis supported the conclusions reached in studies captured by our systematic review [25,29,30]. Previously, a cross-sectional study conducted on 2855 Chinese individuals indicated that serum calcium concentration was inversely associated with the risk of knee OA diagnosed through radiographic means [46]. Although the three studies included in our meta-analysis used different target populations, the causal effect found was similar [25,29,30]. However, Hunter et al. did not find a causal effect between serum calcium levels and OA in female Caucasian twins [47]. Similarly, Zoli et al. found no causal effect between serum calcium levels and hand OA in their case-control study [48]. Due to conflicting conclusions reached by multiple studies, further research into the mechanisms through which calcium has a causal role in OA at both the site and sex level is required. Furthermore, the modifiable nature of mineral status lends itself to major clinical implications in preventing and screening of OA if the correct causal effect is identified.

### 4.3. Low-Density Lipoproteins

The results of our meta-analysis were consistent with the conclusions reached in most studies included in our systematic review [17,18,23]. There was a weak negative causal effect between the reduction in LDL levels and the increase in risk of knee and hip OA found by Funck-Brentano et al. [23]. However, these results contrasted with previously conducted observational studies that suggested increased levels of serum cholesterol were a risk factor for OA [49,50]. It has been previously hypothesized that lipid accumulation in the cartilage of weight-bearing joints could contribute to the pathogenesis of OA [49] and similar results have been shown in experimental animal models [51]. Previous epidemiological studies have reached differing conclusions on the success of statins in preventing OA. Some found that statins lowered risk [52,53], whilst others have found that statins increased risk [54], or found no association [55]. Our findings from the meta-analysis support this alternative hypothesis, that increased LDL levels may serve as a protective effect in lowering the risk of OA diagnosis. There was only a modest OR of protective effect demonstrated for LDL on OA risk relative to the effects seen in education, BMI and smoking so further investigation is required to ascertain whether it is of clinical relevance [18].

### 4.4. Strengths and Limitations

To our knowledge, this systematic review and meta-analysis is the first to evaluate Mendelian randomization studies that assess how different causal risk factors affect the risk of OA, and the first to provide pooled estimates of the causal effect between BMI, serum calcium and LDL on the risk of OA. Prior to this review, causal estimates provided by various independently conducted MR studies, as demonstrated in Figure 2, Figure 7 and Figure 8 for BMI, serum calcium, and LDL, respectively, were heterogeneous and sometimes inconsistent. Moreover, previous observational studies detailed in Section 4.1, Section 4.2 and Section 4.3 provided conflicting associations and biological hypotheses. By providing pooled causal estimates, our review has disambiguated the true extent of the biological effect of lifestyle-related risk factors on OA.

Our quality assessment using STROBE-MR found that 20 out of 23 studies were at a low risk of bias in terms of study design, methodology, and quality of data (Table A1, Appendix B). In addition, no significant publication bias was found when examining the included studies in Figure 9 and Figure 10. Overall, the low risk of methodological and publication bias suggests that our included MR studies are of high quality and is indicative of the robustness of our pooled causal estimates for BMI, serum calcium, and LDL on OA.

In studies utilizing the UK Biobank cohort [16,18,20,21,22,23,24,25,27,29,30,33,34,35,36,37,38], the causal effects represented only the selected European or White-British populations. These were selected predominantly to avoid population stratification. However, these conclusions may not be representative of more general or non-European populations. Similarly, a comparison of conclusions reached across different age demographics may not be feasible. Some studies identified in our meta-analysis utilized older GWAS datasets such as ArcOGEN [19,28,32] that are no longer the most comprehensive available to identify outcome SNPs. More updated datasets, such as those by Zengini et al., should be used [40]. The inclusion of different studies that use overlapping cohorts (particularly UK Biobank), while unavoidable, inevitably reduces the effective sample size and statistical power of our meta-analyses. BMI was the most investigated risk factor in our meta-analysis, but the SNPs used to instrument it varied across cohorts. In some cases, weak causal effects or the absence of any causal effects could not be confirmed since the genetic instruments and sample size used may have been underpowered to detect them [17]. Even when using the MR-Egger method that exploits the assumptions of instrumental variables to estimate pleiotropy, misclassification in these polymorphisms is still possible. Further analyses, utilizing multiple methodologies such as IVW, MR-Egger and median-based tests within a given study will allow the findings to be rigorously tested.

Sub-grouping by other individual-level baseline characteristics such as age, genealogical ancestry and sex may reveal further insight. The lack of sex-stratified GWAS data used in the studies of our systematic review may mean that the sex-specific phenotypic differences remain undetected.

Most of the meta-analyses displayed substantial degrees of heterogeneity and so the conclusions reached should be interpreted with caution despite the usage of random effects models [56]. This heterogeneity can have a greater impact on pooled estimates that report a weak causal effect. The range of meta-analyses that could be conducted was limited by the small number of previous studies that assessed the same causal risk factor. Pooling data on OA at both different sites and diagnosis methods may also have limited our findings as it is clear from our meta-analysis that determinants of OA vary by site. Studies that evaluated hospital-diagnosed OA may have been based on radiographic changes and are therefore likely to be reliable. Self-reported OA, on the other hand, can be unreliable due to its subjective nature and may be subject to recall bias or misclassification that could affect the estimates generated. Furthermore, self-reported cases could indicate other joint pathologies, especially in individuals who have not yet experienced any symptoms of pain but have other symptoms of OA, such as crepitus and swelling. This may exaggerate the reported causal effect.

The Mendelian randomization approach utilizes cumulative lifelong effects of genetic variants and therefore cannot be extrapolated to study the potential effects of clinical treatment [18]. This approximation of the average effect may not be useful in the case of blood mineral levels whose levels vary constantly throughout life [29]. If bidirectional associations are not assessed, reverse causation cannot be completely discounted even if the assumptions for instrumental variable assignment hold. MR approaches aim to remove confounding biases, but it is possible that null results could have been due to misclassification of OA diagnosis [23]. This remains a major challenge for observational and genetic studies of osteoarthritis.

### 4.5. Clinical Implications

Given the moderate causal effect of increasing BMI has on the development of OA, clinical guidelines ought to recommend weight control as a means of primary prevention. Indeed, institutions such as the European Alliance of Associations for Rheumatology and American College of Rheumatology have already recommended ‘weight loss’ as a non-pharmacological means for the self-management of OA [57,58]. We suggest that the next step would be to extend their recommendations to the general population to reduce the population-wide incidence of OA through more aggressive control of BMI in earlier life. Similarly, the moderate causal effect of lower serum calcium on OA would suggest that clinical recommendations pertaining to adequate dietary calcium intake and regular weight-bearing exercise to prevent calcium loss would be useful to the general population to reduce the incidence of OA as primary prevention.

Despite our finding of a weak causal effect of lower LDL on the development of OA, i.e., LDL is a weak protective factor of OA, we do not recommend increasing dietary LDL intake since it is significantly associated with an increased risk of numerous cardiovascular co-morbidities [59].

### 4.6. Future Research

Future research into more quantifiable measures for lifestyle-related risk factors with MR studies such as micronutrient intake of various minerals and vitamins would be useful to examine the impact of various diets and foodstuffs on OA or other musculoskeletal diseases. Furthermore, MR studies on OA from GWAS cohorts of other ethnicities such as East Asians, South Asians, South Americans, etc. would provide insight into how different genetic compositions and different environments/lifestyles stemming from inter-ethnic diversity may result in different magnitudes of causal effect for certain lifestyle-related risk factors on the development of OA.

## 5. Conclusions

Osteoarthritis (OA) is a significant public health issue affecting 303 million people. It is therefore critical that recommendations for management and primary prevention focused on modifiable risk factors are informed by robust research that establishes a causal effect with osteoarthritis, beyond the delimited information of non-causal associations reported by conventional observational studies [60].

Despite the heterogeneous designs and findings of independent MR studies studying the causal effect of various lifestyle-related risk factors on OA, our systematic review and meta-analyses of these MR studies have conclusively demonstrated the extent of the positive causal effect of BMI and that of the negative causal effects of serum calcium and LDL levels on osteoarthritis.

Given the established biological causality between lifestyle-related risk factors and the development of OA, future studies should aim to study how precisely modulating these risk factors with pharmacological and non-pharmacological strategies could modify the incidence and progression of osteoarthritis in various joints as per a dose–response relationship [61,62].

## Figures and Tables

**Table 1:** Study characteristics of all 23 studies included for qualitative analysis.

Study	Year	Age	Ethnicity	Cohort	Design of MR Study	Genetic Instrument	Exposure	Sample Size	Findings
Nicolo-poulos et al. [16]	2020	37–73	White-British	UK Biobank	Two sample. Sample size and cohort from outcome study.	8 variants, CYP1A1/2 (rs2472297), AHR (rs6968554), POR (rs17685), GCKR (rs1260326), EFCAB5 (rs9902453), ABCG2 (rs1481012), MLXIPL (rs7800944), and BDNF (rs6265).	Habitual coffee consumption	*N* = 333,214	Causal effect between habitual coffee consumption and an increased risk of osteoarthrosis, arthropathy and obesity, but some reductions to postmenopausal bleeding.
Hindy et al. [17]	2019	44–73	Swedish	Malmo diet and cancer study, replicated UK Biobank	Two sample. Sample size and cohort from exposure study.	185 lipid associated SNPs. 31 SNPs for BMI. Trait-specific polygenic risk scores for LDL, HDL, cholesterol, triglyceride, BMI, FPG and SBP.	Elevations in traits	Swedish *N* = 27,691, European *N* = 376,435	Causal role of higher LDL cholesterol level in lower risk of diagnosis and higher BMI gives higher risk of OA diagnosis.
Karhunen et al. [18]	2021	41–59	White-British	UK Biobank	Two sample. Sample size and cohort from outcome study.	527 BMI, 78 LDL-C, 197 SBP, 126 smoking and 309 education SNPs.	Traits denoted by SNPs	*N* = 60,800 OA cases and 328,251 controls	Provides evidence supporting protective effects of education and LDL-C and unfavorable effects of BMI and smoking on OA.
Lee et al. [19]	2019	43–75	European	arcOGEN	Two sample. Sample size and cohort from outcome study.	4 SNPs CHRNA3 (rs1051730), SLC25A5P5A9 (rs215614), CHRNB3 (rs6474412), and CYP2B6 (rs7260329).	Smoking	7410 cases, 11,009 controls	Smoking causally associated with reduced risk of OA.
Fan et al. [20]	2021	40–69	European	UK Biobank	Two sample. Sample size and cohort from outcome study.	SNPs for 14 adiponectin, 4 leptin, 4 resistin, 1 chemerin and 1 retinol binding protein 4.	Traits denoted by SNPs	*N* = 50,508	Causal effect between leptin and total OA risk. In addition, adiponectin, leptin and resistin with risk of knee OA.
Zhu et al. [21]	2018	40–69	European	UK Biobank	Two sample. Sample size and cohort from exposure study.	SNPs for BMI and height	BMI and height	*N* = 405,072	BMI had positive risk effects on OA whereas against osteoporosis there was a protective effect. Similar trend for height on same outcomes.
Zhou et al. [22]	2020	40–69	European	UK Biobank	Two sample. Sample size and cohort from outcome study.	2 SNPs for erythrocyte copper.	Blood levels of copper	*N* = 310,999	Copper causally associated with increased risk of OA.
Funck-Brentano et al. [23]	2019	37–76	European	UK Biobank	Two sample. Sample size and cohort from exposure study.	77 BMI, 49 Femoral neck BMD, 48 Lumbar spine BMD, 55 LDL, 71 HDL, 38 Triglycerides, 38 T2D, 25 SBP, 18 CRP SNPs	Traits denoted by SNPs	*N* = 384,838	BMI has a causally effect on OA at weight-bearing joints, but not at the hand. Evidence of causality of all OA, knee OA, and hip OA was also observed for high femoral neck BMD and low systolic BP. However, no evidence of causality for other metabolic factors or CRP level.
He et al. [24]	2021	40–69	European	UK Biobank	Two sample. Sample size and cohort from outcome study.	79 SNPs associated with BMI	Increased BMI	38,472 cases, 424,461 controls	BMI causally associated with OA risk.
Qu et al. [25]	2021	46–54	European	UK Biobank	Two sample. Sample size and cohort from outcome study.	1 b carotene, 7 calcium, 4 iron, 4 phosphorus, 2 retinol, 3 selenium and 3 vitamin E SNPs	Traits denoted by SNPs	*N* = 361,141	Increasing serum calcium levels has a causal effect on reducing OA. Serum retinol levels were inversely associated with hip OA. Evidence for the causal effect of serum calcium, iron and selenium on the risk of OA in women.
Pedersen et al. [26]	2017	20+	Norwegian	Nord-Trøndelag Health Study	One sample	rs1051730 C > T SNP proxy for smoking quantity	Smoking	*N* = 55,745	Smoking casually associated with reduced risk of total joint replacement.
Hyppönen et al. [27]	2019	37–73	White British	UK Biobank	Two sample. Sample size and cohort from exposure study.	Genetic risk score of 76 BMI related variants	Increased BMI	*N* = 337,536	BMI causally associated with OA risk.
Panoutso-poulou et al. [28]	2014	43–75	White British	arcOGEN, Twins UK, Chingford study, Hertfordshire cohort, Nottingham case-control, Genetics of Osteoarthritis and lifestyle study, Tasmanian older adult cohort	One sample	FTO SNP rs8044769, association of rs8044769 with overweight is highly significant (OR[CIs] for allele G = 1.14 [01.08 to 1.19], *p* = 7.5 × 10^−7^).	Obesity	9764 cases, 5362 controls	Causal effect between FTO gene and OA risk exclusively mediated by the effect on BMI.
Zhou et al. [29]	2021	40–69	European	UK Biobank	Two sample. Sample size and cohort from outcome study.	3 iron, 6 calcium, 6 magnesium and 2 copper SNPs	Circulating mineral levels	36,612 cases, 274,387 controls	Zinc and copper status positively associated with OA but not RA.
Zhou et al. [30]	2019	37–73	White British	UK Biobank	Two sample. Sample size and cohort from outcome study.	7 SNPs to proxy Calcium concentration CASR (rs1801725), DGKD (rs1550532), GCKR (rs780094), GATA3 (rs10491003), CARS (rs7481584), DGKH/KIAA0564 (rs7336933), and CYP24A1 (rs1570669)	Serum calcium levels	36,434 cases, 301,101 controls	Decreased risk of osteoarthrosis with increased serum calcium.
Prins et al. [31]	2016	20–90	European	CRP coronary heart disease genetics collaboration	Two sample. Sample size and cohort from outcome study.	2 genetic risk scores, first consisted of 4 SNPs in the CRP gene, second had 18 SNPs for CRP levels.	CRP levels	5755 case, 18,505 controls	10% increase in genetically determined CRP nominally associated with osteoarthritis. May be dependent on BMI and weight gain.
Lee et al. [32]	2018	43–75	European	arcOGEN	Two sample. Sample size and cohort from outcome study.	4 SNPs for coffee consumption neurocalcin delta (NCALD) (rs16868941), cytochrome p450 oxidoreductase (POR) (rs17685), cytochrome p450 family 1 subfamily A member 1 (CYP1A1) (rs2470893), and neuronal cell adhesion molecule (NRCAM) (rs382140)	Habitual coffee consumption	7410 cases, 11,009 controls	Coffee consumption casually associated with increased risk of osteoarthritis.
Hartley et al. [33]	2020	46–54	European	UK Biobank	One sample and two sample analyses completed. Sample size and cohort from exposure study.	IGF1 genetic risk score	IGF1 levels	*N* = 332,092	Serum IGF1 is causally related to higher risk of hip and knee OA.
Bergink et al. [34]	2021	40–69	Icelandic/White British	Iceland/UK Biobank	Two sample. Sample size and cohort from outcome study.	6 SNPs associated with vitamin D levels	Vitamin D levels	41,028 cases, 562,000 controls	Low vitamin D serum levels has no causal effect on the risk of hip or knee OA, unlikely that vitamin D supplementation protects against OA.
Dong et al. [35]	2021	40–69	European	UK Biobank	Two sample. Sample size and cohort from exposure study.	13 SNPSs associated with BMI	Increased BMI	*N* = 452,264	Positive causal effect between childhood BMI and adult osteoarthritis, especially in knee and hip pain
Qu et al. [36]	2020	40–69	European	UK Biobank	Two sample. Sample size and cohort from exposure study.	13 SNPs on circulating sex hormone binding globulin concentration	SHBG concentration	*N* = 361,141	Positive causal effect between circulating SHBG on development of OA and hip OA. Early screening of levels in serum may be useful for clinical assessment.
Cui et al. [37]	2021	40–69	European	UK Biobank	Two sample. Sample size and cohort from outcome study.	35 Type 2 diabetes, 10 fasting glucose and 3 2-h postprandial glucose SNPs	Type 2 diabetes, fasting and 2-h post-prandial glucose	62,892 cases and 596,424 controls	No causality between genetically increased T2D, FG and 2hGlu on OA risk.
Hartley et al. [38]	2020	40–69	European	UK Biobank	One sample and two sample analyses. Sample size and cohort from exposure study.	6 SNPs for BMD, 6 SNPs for BMI	BMD and BMI	*N* = 334,061	BMI-independent causal effect of BMD on hip and knee OA.

## Data Availability

Data can be requested from the corresponding author.

## Appendix A

Search criteria utilized for Medline, returning 446 results:

1. “mendel$ random$”.mp.
2. osteoa$.mp.
3. osteoarthritis.mp. or exp osteoarthritis/
4. ((instrumenta* var* or “covariate” or “covariant” or “causal analysis” or “causal inference”) not MRI).mp.
5. genome wide.mp. or exp genome-wide association study/
6. phenome wide.mp.
7. 2 or 3
8. 1 or 4 or 5 or 6
9. 7 and 8

Search criteria utilized for EMBASE, returning 488 results:

1. “mendel$ random$”.mp.
2. osteoa$.mp.
3. osteoarthritis.mp. or exp osteoarthritis/
4. MR.mp.
5. association.mp. or exp genome-wide association study/
6. exp biological trait/ or exp genetic trait/ or trait.mp.
7. instrumental.mp. or exp instrumental variable analysis/
8. 1 or 4
9. 2 or 3
10. 5 or 6 or 7
11. 8 and
12. 10 and 11

Search criteria utilized for Scopus, returning 642 results:

TITLE-ABS-KEY (“mendel* random*” OR “MR” OR “GWAS” OR “PheWAS” OR “genome wide” OR “phenome wide” OR “genome-wide” OR “phenome-wide”) AND TITLE-ABS-KEY (“osteoarthritis” OR “osteoa*”) AND TITLE-ABS-KEY (“biological trait” OR “genetic trait” OR “trait” OR “instrumental variable” OR “association” OR “instrument”)

## Appendix B

**Table A1:** Quality Assessment tool conducted based on adherence to the Strengthening the Reporting of Mendelian Randomization Studies (STROBE-MR) Guidelines for all 23 studies included in qualitative analysis. Each item is scored between 0 and 1 for each criterion to yield a total score. Upon conversion of the quality assessment score to a percentage, scores of < 75%, 75–85% and > 85% were considered to indicate high, medium and low risk of bias, respectively.

Study and Year of Publication	Title and Abstract	Background	Objectives	Study Design and Data Sources	Statistical Methods: Main Analysis	Software and Pre-Registration	Descriptive Data	Main Results	Sensitivity and Additional Analysis	Key Results	Limitations	Interpretation	Generalizability	MR Core Assumptions	Total Score (out of 14)	% Score
Nicolopoulos et al., 2020 [16]	1	1	1	1	1	1	1	1	0.5	1	1	1	0.5	1	13	92.9
Hindy et al., 2019 [17]	1	1	1	1	1	1	1	1	1	1	1	1	1	1	14	100
Karhunen et al., 2021 [18]	1	1	1	1	1	0.5	1	1	1	1	1	1	0.5	0.5	12.5	89.3
Lee et al., 2019 [19]	1	1	1	1	1	0.5	1	1	1	1	1	1	1	1	13.5	96.4
Fan et al., 2021 [20]	1	1	1	1	1	0.5	0.5	1	1	1	1	1	0.5	1	12.5	89.3
Zhu et al., 2018 [21]	0	1	1	1	1	1	1	1	1	1	0.5	1	0.5	0.5	11.5	82.1
Zhou et al., 2020 [22]	1	1	1	1	1	1	1	1	1	1	1	1	1	1	14	100
Funck-Brentano et al., 2019 [23]	1	1	1	1	1	0.5	1	1	1	1	1	1	0.5	1	13	92.9
He et al., 2021 [24]	1	1	1	1	1	1	1	1	1	1	1	1	0.5	1	13.5	96.4
Qu et al., 2021 [25]	1	1	1	1	1	1	1	1	1	1	1	0.5	0.5	0.5	12.5	89.3
Pedersen et al., 2017 [26]	1	1	1	1	1	1	1	1	1	1	1	1	0.5	1	13.5	96.4
Hyppönen et al., 2019 [27]	1	1	1	1	1	1	1	1	1	1	1	1	1	1	14	100
Panoutsopoulou et al., 2014 [28]	1	1	1	0.5	0.5	0.5	1	1	0	1	1	1	0.5	1	11	78.6
Zhou et al., 2021 [29]	1	1	1	1	1	1	1	1	1	1	1	0.5	0.5	0.5	12.5	8903
Zhou et al., 2019 [30]	1	1	1	1	1	1	1	1	1	1	1	1	1	0.5	13.5	96.4
Prins et al., 2016 [31]	1	1	1	1	1	1	1	1	1	1	1	1	1	1	14	100
Lee et al., 2018 [32]	1	1	1	0.5	1	1	1	1	1	1	1	0.5	0.5	1	12.5	89.3
Hartley et al., 2020 [33]	1	1	1	1	1	1	1	1	1	1	1	1	0.5	1	13.5	96.4
Bergink et al., 2021 [34]	1	1	1	0.5	0.5	1	0.5	1	0	1	1	1	0	0.5	10	71.4
Dong et al., 2021 [35]	1	1	1	1	1	1	1	1	1	1	1	1	1	1	14	100
Qu et al., 2020 [36]	1	1	1	1	1	1	1	1	1	1	1	1	0	0.5	12.5	89.3
Cui et al., 2021 [37]	1	1	1	1	1	1	1	1	1	1	1	1	1	1	14	100
Hartley et al., 2020 [38]	1	1	1	1	1	1	1	1	1	1	1	1	0.5	0.5	13	92.9

**Table A2:** Results of within-study subgroup analyses and OA diagnosis information.

Author	Year	Exposure	Osteoarthritis Diagnosis	Subgroup Analyzed	Estimates (Odds Ratio)	Upper CI	Lower CI
Nicolopoulos	2020	Coffee	Joint Replacement + Radiographic (Hip and Knee)	All	1.23	1.35	1.11
Hindy	2019	LDL cholesterol	Self-reported + Joint Replacement + Clinician diagnosed	Clinician Diagnosed	MR-Egger 0.83	0.95	0.73
				All	0.87	0.98	0.78
		BMI		Clinician Diagnosed	1.65	2.41	1.14
					MR Egger 3.25 (presence of pleiotropic bias)	8.39	1.26
				Joint Replacement	MR Egger 3.81	10.4	1.39
				All	MR Egger 3.41	8.15	1.43
Karhunen	2021	Education	Self-reported + Clinician diagnosed (Hip and Knee)	All	0.59	0.64	0.54
		LDL-C			0.94	0.98	0.91
		BMI			1.82	1.92	1.73
		Smoking			2.23	2.68	1.85
		SBP			0.98	0.9	1.06
Lee	2019	Smoking	Joint replacement + Radiographic	All	IVW β = −0.056, SE = 0.027, *p* = 0.035, intercept = −0.005; *p* = 0.848		
					MR Egger β = −0.048, SE = 0.048, *p* = 0.427		
					W-Med β = −0.056, SE = 0.028, *p* = 0.046		
Fan	2021	Leptin	Self-reported + Clinician diagnosed (hospital records)	All	IVW 2.40/W-Med 2.94	5.09/6.99	1.13/1.23
		Adiponectin		Knee Only	IVW 1.28	1.61	1.01
		Resistin			IVW 3.44	10.03	1.18
		Leptin			IVW 1.18	1.36	1.03
Zhu	2018	BMI	Self-reported + Clinician diagnosed (hospital records)	All	1.5		
		Height			1.09		
Zhou	2020	Erythrocyte copper	Self-reported + Clinician diagnosed (hospital records)	All	1.07	1.13	1.02
Funck-Brentano	2019	BMI	Self-reported + Clinician diagnosed (hospital records) + Joint replacement	All	1.57	1.71	1.44
				Knee Only	1.76	1.99	1.56
				Hip Only	1.52	1.78	1.31
				Hand Only	1	1.31	0.76
		Femoral neck BMD		All	1.14	1.22	1.06
				Knee Only	1.18	1.32	1.05
				Hip Only	1.22	1.35	1.09
				Hand Only	1.11	1.39	0.88
		Lumbar spine BMD		Knee Only	1.15	1.26	1.06
		SBP		All	0.64	0.77	0.54
				Knee Only	0.66	0.77	0.57
				Hip Only	0.63	0.82	0.48
		BMI		Knee Replacement Only	2.3	2.75	1.93
				Hip Replacement Only	1.65	1.92	1.41
		Femoral neck BMD		Knee Replacement Only	1.27	1.48	1.09
				Hip Replacement Only	1.17	1.31	1.05
		SBP		Knee Replacement Only	0.64	0.83	0.5
				Hip Replacement Only	0.64	0.85	0.48
He	2021	BMI	Joint replacement + Radiographic (Hip and Knee)	All	IVW 1.028	1.036	1.021
					W-Med 1.028	1.037	1.019
					MR Egger 1.028, intercept 1.3 × 10^−5^, *p* = 0.959	1.046	1.009
Qu	2021	Serum calcium	Joint replacement + Radiographic	All	0.712	0.85	0.595
				Hip Only	0.531	0.799	0.352
				Knee Only	0.645	0.901	0.461
		Serum retinol		Hip Only	0.447	0.778	0.257
		Serum calcium		Female Only	0.967	0.998	0.936
		Serum iron			1.006	1.012	1
		Serum selenium			0.96	0.999	0.923
				Male Only	0.953	0.994	0.914
Pedersen	2017	Smoking quantity	Joint replacement	N/A	0.97	0.98	0.97
		Current smokers			0.84	0.98	0.76
		Former smokers			0.97	1.07	0.88
		Never smokers			0.97	1.06	0.89
Zhou	2019	BMI	Clinician diagnosed (hospital records)	N/A	IVW 1.52	1.68	1.37
					W-Med 1.42	1.61	1.25
					W-Mod 1.40	1.63	1.2
					MR Egger 1.43	1.83	1.11
					MR PRESSO 1.50	1.65	1.37
Panoutsopoulou	2014	FTO/BMI	Joint replacement + Radiographic	All	1.14	1.19	1.08
Zhou	2021	Copper	Clinician diagnosed (hospital records)		1.07	1.13	1.02
		Zinc			1.07	1.13	1.01
		Copper		Localized OA	1.08	1.15	1.03
		Zinc		Generalized OA	1.18	1.31	1.05
				Unspecified OA	1.21	1.31	1.11
		Calcium		Localized OA	0.83	0.98	0.69
		Iron		Male Only—Unspecified OA	1.27	1.54	1.05
		Calcium		Male Only—All	0.67	0.98	0.46
				Male Only—Generalized OA	0.35	0.93	0.13
Zhou	2019	Calcium	Joint replacement + Radiographic (Hip and Knee)	Osteoarthrosis	IVW 0.67	0.88	0.51
					W-Med 0.63	0.85	0.47
					W-Mod 0.62	0.85	0.45
					MR Egger 0.55	0.91	0.33
				Hip and Knee Only	IVW 0.34	0.7	0.17
					W-Med 0.31	0.73	0.14
					W-Mod 0.31	0.76	0.13
					MR Egger 0.29	1.1	0.08
Prins	2016	CRP serum	Joint replacement + Radiographic (Hip and Knee) + Clinician diagnosed (hospital records) + Self-reported	Knee Only	1.17	1.36	1.01
Lee	2018	Coffee	Joint replacement + Radiographic (Hip and Knee)	All	IVW β = 0.381	SE = 0.17 *p* =0.025	
					W-Med β = 0.419	SE = 0.206, *p* = 0.047	
					MR Egger β = −0.518	SE = 1.270, *p* = 0.723	
				Knee Only	IVW β = 0.451	SE = 0.227, *p* = 0.047	
				Hip Only	IVW β = 0.326	SE = 0.23, *p* = 0.156	
Hartley	2020	IGF1	Clinician diagnosed (Hospital records)	Hip Only	1.57	2.01	1.21
				Knee Only	1.3	1.58	1.07
				Hand Only	0.98	1.7	0.57
		IGF1/no BMI		Hip Only	1.32	1.58	1.09
				Knee Only	1.14	1.31	0.99
Bergink	2021	Vitamin D	Clinician diagnosed (Hospital records)	Knee Only	1.03	1.26	0.84
				Hip Only	1.06	1.35	0.83
Dong	2021	BMI	Self-reported + Clinician diagnosed (Hospital records)	N/A	1.07	1.1	1.05
Qu	2020	Sex hormone binding globulin	Joint replacement + Radiographic (Hip and Knee)	All	1.086	1.168	1.009
				Hip Only	1.423	1.66	1.219
Cui	2021	Type 2 diabetes	Joint replacement + Radiographic (Hip and Knee) + Clinician diagnosed (hospital records) + Self-reported	Hip Only	MR Egger 1.1708	1.4476	0.9469
				Knee Only	MR Egger 0.9046	1.1085	0.788
		Fasting glucose		Hip Only	MR Egger 0.4634	1.1617	0.1848
				Knee Only	MR Egger 0.589	1.0943	0.3697
		2-h postprandial glucose			MR Egger 1.3062	2.819	0.254
				Hip Only	MR Egger 1.3652	2.5993	0.7171
Hartley	2020	BMD	Clinician diagnosed (hospital records) + Radiographic + Self-reported	Hip Only	1.28	1.57	1.05
				Knee Only	1.4	1.63	1.2
